# An open-source fine-tuned large language model for radiological impression generation: a multi-reader performance study

**DOI:** 10.1186/s12880-024-01435-w

**Published:** 2024-09-27

**Authors:** Adrian Serapio, Gunvant Chaudhari, Cody Savage, Yoo Jin Lee, Maya Vella, Shravan Sridhar, Jamie Lee Schroeder, Jonathan Liu, Adam Yala, Jae Ho Sohn

**Affiliations:** 1 Department of Radiology and Biomedical Imaging, University of California, San Francisco, San Francisco, CA USA; 2 Department of Radiology, University of Maryland Medical Center, Baltimore, MD USA; 3 Department of Radiology, University of Washington, Seattle, WA USA; 4 MedStar Georgetown University Hospital, Washington, DC USA; 5 Computational Precision Health, University of California, Berkeley and University of California, San Francisco, Berkeley, USA

**Keywords:** Natural language processing, Large language model, Open-source, Summarization, Impressions

## Abstract

**Background:**

The impression section integrates key findings of a radiology report but can be subjective and variable. We sought to fine-tune and evaluate an open-source Large Language Model (LLM) in automatically generating impressions from the remainder of a radiology report across different imaging modalities and hospitals.

**Methods:**

In this institutional review board-approved retrospective study, we collated a dataset of CT, US, and MRI radiology reports from the University of California San Francisco Medical Center (UCSFMC) (*n* = 372,716) and the Zuckerberg San Francisco General (ZSFG) Hospital and Trauma Center (*n* = 60,049), both under a single institution. The Recall-Oriented Understudy for Gisting Evaluation (ROUGE) score, an automatic natural language evaluation metric that measures word overlap, was used for automatic natural language evaluation. A reader study with five cardiothoracic radiologists was performed to more strictly evaluate the model’s performance on a specific modality (CT chest exams) with a radiologist subspecialist baseline. We stratified the results of the reader performance study based on the diagnosis category and the original impression length to gauge case complexity.

**Results:**

The LLM achieved ROUGE-L scores of 46.51, 44.2, and 50.96 on UCSFMC and upon external validation, ROUGE-L scores of 40.74, 37.89, and 24.61 on ZSFG across the CT, US, and MRI modalities respectively, implying a substantial degree of overlap between the model-generated impressions and impressions written by the subspecialist attending radiologists, but with a degree of degradation upon external validation. In our reader study, the model-generated impressions achieved overall mean scores of 3.56/4, 3.92/4, 3.37/4, 18.29 s,12.32 words, and 84 while the original impression written by a subspecialist radiologist achieved overall mean scores of 3.75/4, 3.87/4, 3.54/4, 12.2 s, 5.74 words, and 89 for clinical accuracy, grammatical accuracy, stylistic quality, edit time, edit distance, and ROUGE-L score respectively. The LLM achieved the highest clinical accuracy ratings for acute/emergent findings and on shorter impressions.

**Conclusions:**

An open-source fine-tuned LLM can generate impressions to a satisfactory level of clinical accuracy, grammatical accuracy, and stylistic quality. Our reader performance study demonstrates the potential of large language models in drafting radiology report impressions that can aid in streamlining radiologists’ workflows.

## Introduction

Radiology reports synthesize a radiologist’s interpretations which are essential in communicating the current condition of a patient [1]. Radiology reports typically consist of an exam type, clinical history, comparison, technique, radiation dose, findings, and impression section [2]. The impression section is of utmost importance, as it summarizes the key findings of the radiology report and carries the most weight in influencing the clinical decision-making of the consulting physician [3, 4]. As it stands, the process of generating the impression section is not always standardized and can be subjective [5]. Automatically generating impressions can help to ensure that essential findings are not omitted while also keeping the impressions succinct.

Since the Large Language Models (LLMs) ChatGPT and GPT-4 were released in November 2022 and March 2023 respectively, multiple studies have shown how these LLMs could be applied to a variety of radiological tasks such as structured reporting, question answering on a radiology board-style examination, and response to common lung cancer questions [6–8]. Closely related to our work, GPT-4 was shown to generate impressions for radiology reports [9].

Given that ChatGPT and GPT-4 are close-sourced models only available via web APIs, we believe that it is the crucial next step to clinically validate the performance of fine-tuned open-source large language models, enhancing access and replicability that will greatly aid future development in this area. Especially for private clinical datasets, open-source models provide the advantage of eliminating the need to upload sensitive patient data to a cloud service and instead be trained and deployed locally [10].

In this study, our objective was to evaluate the performance of a fine-tuned open-source LLM in generating impressions to summarize radiology reports over multiple imaging modalities and hospitals which would test the model’s capacity to generalize across different settings. We aimed to evaluate the fine-tuned model’s performance through a clinical reader performance study on a specific modality with subspecialty radiologists.

## Methods

### Datasets and Corpora

The radiology reports in this study were retrospectively collected with the University of California San Francisco’s Institutional Review Board approval and informed consent waiver, following the Helsinki Declaration of 1975, as revised in 2013. All methods were performed in accordance with the relevant guidelines and regulations. We gathered CT, US, and MRI reports from two hospitals under one institutional affiliation. The University of California San Francisco Medical Center (UCSFMC) is an academic tertiary referral center, while the Zuckerberg San Francisco General Hospital (ZSFG) and Trauma Center is a level-1 trauma center and county safety net hospital. A total of 372,716 radiology reports between January 1, 2021 and October 22, 2022 were consecutively and comprehensively sourced from UCSFMC, while a total of 60,049 radiology reports between January 1, 2022 and December 29, 2022 were consecutively and comprehensively sourced from ZSFG. In terms of reporting style, both UCSFMC and ZSFG follow structured reporting. Moreover, both hospitals utilize a system where reports are initially prepared by residents and then reviewed and finalized by attending radiologists, who provide revisions before signing off. As such, all reports reflect the work and approval of the attending radiologist. Table 1 summarizes the demographics of the datasets sourced from UCSFMC and ZSFG.

**Table 1:** Characteristics of the UCSFMC training, validation, and test sets and the ZSFG independent test dataset

Characteristic	UCSFMC Training set (*n* = 282,525)	UCSFMC Validation set (*n* = 35,631)	UCSFMC Test set (*n* = 35,124)	ZSFG independent test set (*n* = 59,923)
Age (y)	51.19 ± 22.84	50.66 ± 22.75	51.22 ± 22.89	52.62 ± 19.31
Sex (%)				
Male	128,235 (45.39)	16,444 (46.15)	16,009 (45.58)	32,137 (53.63)
Female	153,952 (54.49)	19,150 (53.75)	19,077 (54.31)	27,760 (46.33)
Other	338 (0.12)	37 (0.10)	38 (0.11)	26 (0.04)
Imaging modality (%)				
CT	119,600 (42.33)	15,060 (42.27)	14,810 (42.16)	36,640 (61.14)
MRI	84,939 (30.06)	10,735 (30.13)	10,691 (30.44)	7578 (12.65)
US	77,986 (27.60)	9836 (27.60)	9623 (27.40)	15,705 (26.21)
Patient status (%)				
Outpatient	182,829 (64.71)	22,801 (64)	22,807 (64.93)	24,157 (40.31)
Inpatient	59,129 (20.93)	7614 (21.37)	7401 (21.07)	14,872 (34.69)
Emergency	33,913 (12)	4353 (12.21)	4154 (11.83)	20,790 (24.82)
Other	6654 (2.36)	863 (2.42)	762 (2.17)	104 (0.18)
Stat (%)				
Non-stat	282,404 (99.96)	35,614 (99.95)	35,105 (99.95)	35,968 (60.03)
Stat	121 (0.04)	17 (0.05)	19 (0.05)	23,955 (39.97)
Body part imaged (%)				
Abdomen/Pelvis	72,284 (25.59)	9143 (25.66)	9069 (25.82)	16,090 (26.85)
Brain	51,019 (18.06)	6476 (18.18)	6258 (17.82)	12,233 (20.42)
Chest	38,497 (13.63)	4840 (13.58)	4788 (13.64)	9182 (15.32)
Spine	23,861 (8.45)	3067 (8.61)	3067 (8.73)	2222 (3.71)
Neck	13,340 (4.72)	1691 (4.75)	1636 (4.66)	3117 (5.20)
Renal/Kidney	11,936 (4.22)	1484 (4.16)	1446 (4.12)	1370 (2.29)
Extremity	11,496 (4.07)	1435 (4.03)	1462 (4.16)	1230 (2.05)
Prostate	3763 (1.33)	495 (1.39)	464 (1.32)	0 (0)
Breast	3277 (1.16)	390 (1.09)	430 (1.22)	7 (0.01)
Knee	3260 (1.15)	391 (1.1)	399 (1.14)	407 (0.68)
Liver	2537 (0.9)	320 (0.9)	331 (0.94)	1077 (1.8)
Hip	2251 (0.8)	299 (0.84)	271 (0.77)	124 (0.21)
Heart	1442 (0.51)	193 (0.54)	156 (0.44)	0 (0)
Head	407 (0.14)	76 (0.21)	68 (0.19)	1098 (1.83)
Other	43,155 (15.27)	5331 (14.96)	5279 (15.03)	11,766 (19.63)

**Fig. 1:**
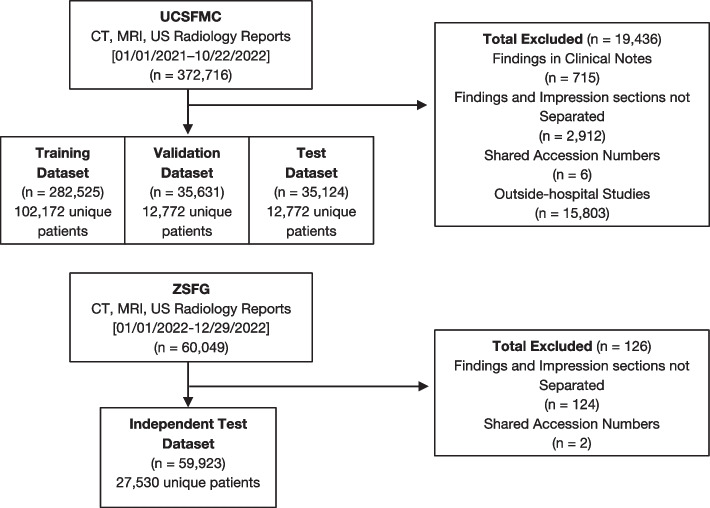
Inclusion and exclusion of data. The UCSFMC dataset was used for training, validation, and testing, and was randomized by patient. The ZSFG dataset was used as an independent test set for external validation

We excluded all outside hospital imported cases as they did not have associated radiology reports in the system, reports with findings stored in clinical notes, reports that did not separate the findings and impression section, and reports that shared the same accession numbers. From UCSFMC, a total of 19,436 reports were excluded, leaving 353,280 reports that were used in our study. 102172, 12772, and 12772 patients were assigned for training, validation, and testing respectively. This resulted in training, validation, and test datasets composed of 282525, 35631, and 35124 reports respectively. From ZSFG, a total of 126 reports were excluded which resulted in an independent test set of 59923 reports from 27530 patients (Fig. 1).

### Model development

**Fig. 2:**
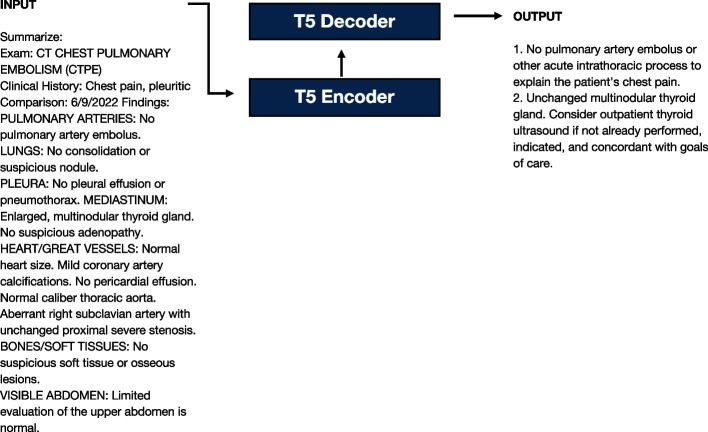
Model architecture. The Text-to-Text Transformer (T5) is an encoder-decoder architecture that takes in input text sequences and outputs text sequences. The exam type, clinical history, comparison, and findings sections are fed into a tokenizer and passed into the encoder while the impression section is fed into a tokenizer and subsequently passed into the decoder block for model training

We fine-tuned the open-source Text-to-Text Transformer (T5) large language model to generate impressions [11]. The T5 is an instruction-tuned model that has been initially pre-trained on the colossal, cleaned version of Common Crawl’s web crawl corpus (C4) dataset, composed of websites scraped from the internet [12]. The remainder of each radiology report excluding the impression serves as the input text and the impression section of each radiology report serves as the output text, where both sequences are tokenized and then subsequently fed into the model (Fig. 2). PyTorch (version 2.1.0) and the HuggingFace transformers library (version 4.35.0) were used to implement these methods [13, 14]. We used the AdamW optimizer with a learning rate of 0.0003, a batch size of 4, and accumulated grad batches of 32 for an effective batch size of 128 [15]. All code is available at https://github.com/bdrad/radiological-report-impression-generation.

### Automated lexical evaluation metrics

The Recall-Oriented Understudy for Gisting Evaluation (ROUGE) score, the standard performance metric for automated text summarization, was calculated to evaluate the models’ performance in impression generation [16]. ROUGE-1 and ROUGE-2 measure the overlap of a unigram and bigrams, respectively, between the original impression and generated impression. On the other hand, the ROUGE-L is based on the longest common subsequence, measuring sentence-level semantic similarity. A higher ROUGE score indicates a higher-quality summary with a maximum ROUGE score of 100. We calculated the ROUGE-1, ROUGE-2, and ROUGE-L scores over the UCSFMC test dataset and the ZSFG independent test dataset.

### Clinical reader performance study

We conducted a reader performance study with five board-certified cardiothoracic radiologists who have eight, seven, six, eight, and six years of experience (inclusive of residency and fellowship training). The study involved 60 CT chest reports from 60 unique patients that were sampled from the UCSFMC test dataset. The sample size was determined by the time and resources required to have attending radiologists manually evaluate and edit impressions. Furthermore, we confirmed a similar size in Sun et al. who have previously conducted a reader study for automatic impression generation based on 50 reports and limited the evaluation to the modality of Chest X-rays [9]. We focused our reader study on evaluating Chest CTs to impose a more stringent and granular analysis of the errors of generated impressions when compared to a subspecialist cardiothoracic radiologist baseline.

Forty of the reports were randomly selected to show the generated impression, while 20 show the original impression written by the attending thoracic radiologist. This reader performance study structure involving both model-generated and radiologist's final impressions was chosen for better evaluation of the LLM, including any of its potential errors or unexpected behaviors. We note that the CT scan images were not provided to the radiologists.

Each radiologist was asked to rate the impression in terms of clinical accuracy, grammatical accuracy, and stylistic quality. They may optionally edit the impression. Edit time and edit distance (number of words changed) were recorded to quantitatively measure workflow efficiency. We also calculate the ROUGE scores of the original or generated impression with respect to the radiologist edits. We note, however, that this score cannot be directly compared to the previously calculated ROUGE scores, as the previous one was subject against a separately written original impression, while in this case, measuring against an edited impression by a reader.

We also stratified the complexity of the reports in the reader study according to diagnostic categories and the length of the original impression. To determine each study’s diagnosis category, a thoracic radiologist with eight years of experience who did not participate in the reader performance study examined the clinical history and original impression of each report. The radiologist defined it into the following categories: Cancer staging, Acute/emergent findings, Interstitial lung disease, Nodules, Lung Transplant, and Aneurysm. For model evaluation, the Interstitial lung disease, Nodules, Lung Transplant, and Aneurysm were consolidated into a single “Other” category. In terms of impression length, each of the original impressions was classified into three categories: Short, Medium, and Long. The reports were sorted by original impression length with short, medium, and long corresponding to the bottom 20, middle 20, and top 20 reports in terms of original impression word length.

### Statistical analysis

A Mann–Whitney U test was used to calculate the *P* values comparing the ratings for the model-generated impressions and the original impressions written by an attending radiologist in terms of clinical accuracy, grammatical accuracy, stylistic quality, edit time, and edit distance [17]. 95% CIs were generated for the ROUGE scores and reader performance study metrics using bootstrapping with resampling. A multi-rater intraclass correlation was computed to measure inter-rater variability for the ordinal clinical metrics of clinical accuracy, grammatical accuracy, and stylistic quality as applicable [18]. All statistical analysis was conducted in Python 3.10.9 using the Numpy (version 1.26.4) Scipy (version 1.11.1), and Pingouin (version 0.5.4) packages [19–21].

## Results

### Dataset characteristics

For UCSFMC, we excluded 15803 reports that were non-reportable due to being outside-hospital studies, 715 reports with findings stored in clinical notes, 2912 reports that did not separate the findings and impression section, and 6 reports that share the same accession numbers. For ZSFG, we excluded 124 reports that did not separate the findings and impression section and 2 reports that share the same accession numbers (Fig. 1).

After dataset exclusion, we tabulate the age, sex, imaging modality, status (Emergency/Inpatient/Outpatient), stat (Is Stat/Non-stat), and body part imaged for the UCSFMC training, validation, test datasets and ZSFG independent test dataset (Table 1). In addition to the demographics of the 60 CT chest reports used in the reader performance study, Table 2 documents the stratifications by diagnosis category and original impression length to gauge case complexity.

**Table 2:** Characteristics of CT chest cases used in the reader study evaluation dataset assigned for model-generated and radiologist-written impression evaluation

Characteristic	Model-generated Cases (*n* = 40)	Radiologist-written Cases (*n* = 20)
Age (y)	58.48 ± 21.93	53.55 ± 23
Sex		
Male	18 (45)	10 (50)
Female	22 (55)	10 (50)
Study type		
CT chest without contrast	19 (42.5)	9 (45)
CT chest with contrast	9 (22.5)	4 (20)
CT chest pulmonary embolism	6 (15)	2 (10)
CT chest high resolution	3 (7.5)	4 (20)
CT chest with contrast (PETCT)	3 (7.5)	1 (5)
CT chest without contrast (PETCT)	2 (5)	0 (0)
Diagnosis category		
Cancer staging	16 (40)	6 (35)
Acute/emergent	15 (37.5)	7 (30)
Interstitial lung disease	5 (12.5)	2 (10)
Nodules	4 (10)	2 (10)
Lung transplant	0 (0)	2 (10)
Aneurysm	0 (0)	1 (5)
Original impression length		
Short (L < = 27 words)	14 (35)	7 (35)
Medium (28 < L < = 45 words)	13 (32.5)	7 (35)
Long (L > = 46 words)	13 (32.5)	6 (30)

### Automated lexical evaluation metrics

Table 3 depicts the automated lexical metrics achieved by the large language model on both the UCSFMC and ZSFG test datasets. The ROUGE-1, ROUGE-2, and ROUGE-L scores quantify the overall adherence of large language models in generating impressions to the level of the finalized impressions written by attending radiologists. The large language model achieved a ROUGE-1 score of 53.22 (95% CI: 52.88, 53.62), ROUGE-2 score of 51.26 (95% CI: 50.87, 51.65), and ROUGE-L score of 46.51 (95% CI: 46.13, 46.89) on the CT modality for the UCSFMC test dataset. The model achieved a slightly lower ROUGE-1 score of 46.57 (95% CI: 46.37, 46.79), ROUGE-2 score of 31.87 (95% CI: 31.65, 32.09), and ROUGE-L score of 40.74 (95% CI: 40.52, 40.93) on the CT modality for the ZSFG independent test dataset. We observe a degree of degradation in model quality when externally validated for the CT modality.

**Table 3:** Summary statistics for the automated lexical ROUGE scores results of the large language model on the UCSFMC test dataset and ZSFG independent test set over multiple imaging modalities

Modality	ROUGE-1	ROUGE-2	ROUGE-L
CT			
UCSFMC test dataset	53.22 (52.88, 53.62)	51.26 (50.87, 51.65)	46.51 (46.13, 46.89)
ZSFG independent test dataset	46.57 (46.37, 46.79)	31.87 (31.65, 32.09)	40.74 (40.52, 40.93)
MRI			
UCSFMC test dataset	51.26 (50.87, 51.65)	35.36 (34.91, 35.79)	44.2 (43.78, 44.65)
ZSFG independent test dataset	45.04 (44.59, 45.5)	29.47 (29, 29.95)	37.89 (37.43, 38.31)
US			
UCSFMC test dataset	56.41 (55.89, 56.9)	41.15 (40.54, 41.76)	50.96 (50.46, 51.48)
ZSFG independent test dataset	32 (31.75, 32.24)	13.87 (13.65, 14.08)	24.61 (24.38, 24.85)

The large language model achieved a ROUGE-1 score of 51.26 (95% CI: 50.87, 51.65), ROUGE-2 score of 35.36 (95% CI: 34.91, 35.79), and ROUGE-L score of 44.2 (95% CI: 43.78, 44.65) on the MRI modality for the UCSFMC test dataset. The model achieved a slightly lower ROUGE-1 score of 45.04 (95% CI: 44.59, 45.5), ROUGE-2 score of 29.47 (95% CI: 29, 29.95), and ROUGE-L score of 37.89 (95% CI: 37.43, 38.31) on the MRI modality for the ZSFG independent test dataset. Similarly, we observe a degree of degradation in model quality when externally validated for the MRI modality.

The large language model achieved a ROUGE-1 score of 56.41 (95% CI: 55.89, 56.9), ROUGE-2 score of 41.15 (95% CI: 40.54, 41.76), and ROUGE-L score of 50.96 (95% CI: 50.46, 51.48) on the US modality for the UCSFMC test dataset. The model achieved a lower ROUGE-1 of 32 (95% CI: 31.75, 32.24), ROUGE-2 score of 13.87 (95% CI: 13.65, 14.08), and ROUGE-L score of 24.61 (95% CI: 24.38, 24.85) on the US modality for the ZSFG independent test dataset. Similarly, we observe a greater degree of degradation in model quality when externally validated for the US modality.

### Clinical reader performance study

The model achieved an overall mean clinical accuracy of 3.56 (3.46, 3.67) out of 4, grammatical accuracy of 3.92 (3.89, 3.96) out of 4, and stylistic quality of 3.37 (3.26, 3.47) out of 4, edit time of 18.29 (14.85, 21.98) seconds, and edit distance of 12.32 (9.88, 14.97) words. The radiologist baseline, which was the original cardiothoracic radiologist’s impression, achieved an overall mean clinical accuracy of 3.75 (3.61, 3.88) out of 4, grammatical accuracy of 3.87 (3.79, 3.94) out of 4, and stylistic quality of 3.54 (3.42, 3.65) out of 4, edit time of 12.2 (8.48, 16.48) seconds, and edit distance of 5.74 (4.06, 7.72) words (Table 4). Moreover, with respect to the edited impressions, the model-written impressions achieved a mean ROUGE-1, ROUGE-2, and ROUGE-L scores of 85 (82.89, 88.22), 81 (77.04, 84.41), and 84 (80.72, 87.13) respectively. On the other hand, the original impressions written by an attending radiologist achieved mean scores of 89 (85.96, 92.69), 85 (76.90, 89.30), and 89 (84.76, 92.31) respectively (Table 5).

**Table 4:** Statistics of the results of the reader performance study along with stratifications based on the diagnosis category and original impression length

Parameter	Clinical Accuracy (out of 4) ↑	Grammatical Accuracy (out of 4) ↑	Stylistic Quality (out of 4) ↑	Edit Time (in seconds) ↓	Edit Distance (in words) ↓
Overall					
LLM	3.56 (3.46, 3.67)	3.92 (3.89, 3.96)	3.37 (3.26, 3.47)	18.29 (14.85, 21.98)	12.32 (9.88, 14.97)
Radiologist	3.75 (3.61, 3.88)	3.87 (3.79, 3.94)	3.54 (3.42, 3.65)	12.2 (8.48, 16.48)	5.74 (4.06, 7.72)
*P*-value	.009	.15	.08	.13	.003
Diagnosis category					
Cancer staging					
LLM	3.59 (3.41, 3.74)	3.92 (3.86, 3.98)	3.35 (3.19, 3.49)	22.22 (15.88, 29.15)	12.75 (9.26, 16.59)
Radiologist	3.67 (3.36, 3.9)	3.83 (3.7, 3.97)	3.53 (3.37, 3.7)	16.34 (8.55, 25.42)	8.43 (4.73, 13.43)
Acute/Emergent					
LLM	3.64 (3.45, 3.8)	3.96 (3.91, 4)	3.49 (3.33, 3.64)	10.94 (7.39, 14.79)	8.39 (5.57, 11.57)
Radiologist	3.71 (3.46, 3.91)	3.86 (3.71, 3.97)	3.37 (3.17, 3.57)	11.62 (6.43, 17.93)	6.63 (3.89, 9.8)
Other^a^					
LLM	3.4 (3.16, 3.62)	3.87 (3.73, 3.98)	3.18 (2.93, 3.4)	23.55 (15.73, 32)	18.11 (11.2, 25.73)
Radiologist	3.86 (3.66, 4)	3.91 (3.83, 4)	3.71 (3.54, 3.86)	9.24 (3.83, 17.11)	2.54 (1.26, 4.06)
Original impression length					
Short (L < 27 words)					
LLM	3.66 (3.47, 3.81)	3.89 (3.79, 3.96)	3.37 (3.2, 3.54)	21.66 (14.95, 29.2)	15.07 (10.29, 20.33)
Radiologist	3.77 (3.49, 3.97)	3.89 (3.74, 4)	3.63 (3.46, 3.8)	10.25 (4.88, 16.5)	5.66 (3, 8.83)
Medium (28 < L < = 45 words)					
LLM	3.45 (3.23, 3.63)	3.94 (3.88, 3.98)	3.25 (3.05, 3.43)	16.32 (11.3, 21.93)	13.97 (9.28, 19.18)
Radiologist	3.66 (3.37, 3.89)	3.89 (3.77, 3.97)	3.37 (3.14, 3.57)	14.87 (8.74, 22.26)	7.31 (4.06, 11.63)
Long (L > = 46 words)					
LLM	3.58 (3.38, 3.75)	3.95 (3.89, 4)	3.48 (3.32, 3.62)	16.63 (11.12, 22.57)	7.71 (5.52, 10.06)
Radiologist	3.83 (3.6, 4)	3.83 (3.7, 3.97)	3.63 (3.47, 3.8)	11.36 (4.82, 20.58)	4 (2.13, 6.1)

↑ indicates that higher is better and ↓ indicates that lower is better

^a^We combined cases that depicted interstitial lung disease, nodules, lung transplant, and aneurysm into a single other category

**Table 5:** ROUGE score summary statistics from the reader performance study measuring the overlap between the impression being evaluated and the revised impression written by the attending radiologist reader

Parameter	ROUGE-1	ROUGE-2	ROUGE-L
Overall			
LLM	85 (82.89, 88.22)	81 (77.04, 84.41)	84 (80.72, 87.13)
Radiologist	89 (85.96, 92.69)	85 (79.60, 89.30)	89 (84.76, 92.31)
* P*-value	0.17	0.23	0.16

Table 4 also depicts mean scores of the model-generated and radiologist-written impressions stratified by diagnosis category and original impression length. For reports that contained acute/emergent findings, the LLM achieved the highest clinical accuracy rating of 3.64 (3.45, 3.8) out of 4, whereas the radiologist baseline achieved a clinical accuracy of 3.71 (3.46, 3.91) out of 4. The model slightly underperforms in the category “Other” (Interstitial Lung Disease, Nodules, and Lung Transplant) achieving a clinical accuracy rating of 3.4 (3.16, 3.62) out of 4, while the radiologist baseline achieves a clinical accuracy of 3.86 (3.66, 4) out of 4. In terms of impression length, the LLM performs the best in clinical accuracy on shorter impressions achieving a clinical accuracy rating of 3.66 (3.47, 3.81) out of 4 in this category, and slightly underperforms in longer impressions achieving a clinical accuracy rating of 3.45 (3.23, 3.63) out of 4 and 3.58 (3.38, 3.75) in the Medium and Long categories.

Multi-rater interclass correlation scores were calculated to measure the inter-rater reliability of the group of radiologists who participated in the reader performance study. Given the limited variance of the grammatical accuracy metric (σ^2^ = 0.098) as opposed to the clinical accuracy (σ^2^ = 0.58) and stylistic quality (σ^2^ = 0.47), we chose to report intra-class correlations for clinical accuracy and stylistic quality given the limited ability of the intraclass correlation score to quantify agreement over limited variance [18]. The level of agreement among the readers was moderate for both metrics with ICC scores of 0.67 and 0.57 for clinical accuracy and stylistic quality respectively.

### Error analysis

**Fig. 3:**
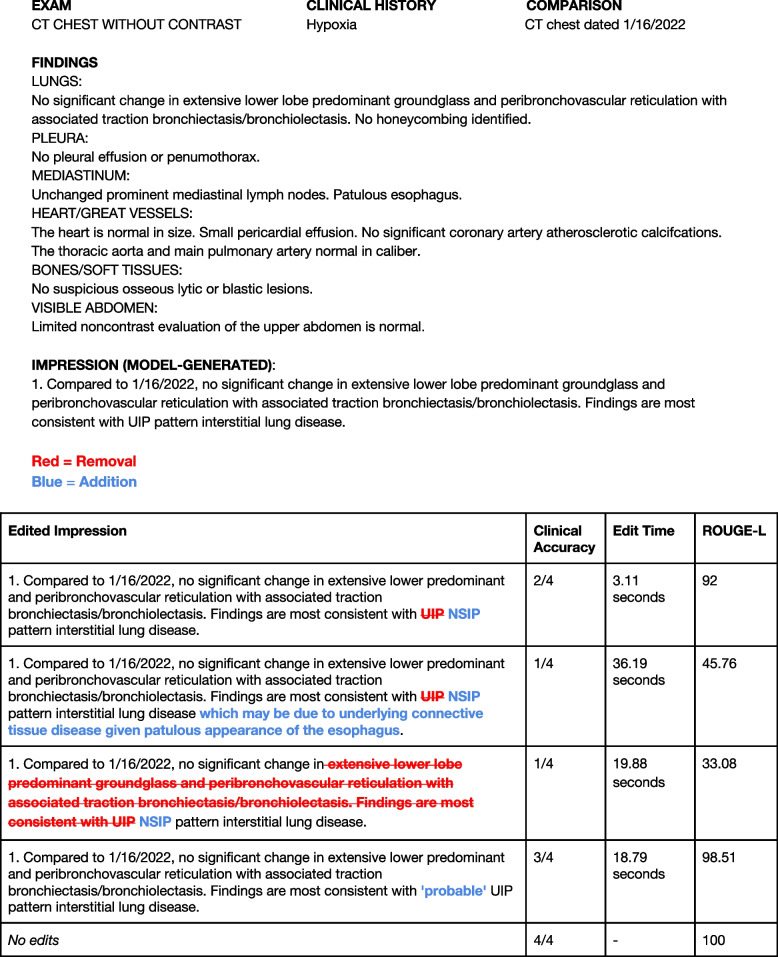
Lowest-scoring model-generated impression in terms of clinical accuracy. The lower-scoring model generated impression in terms of clinical accuracy and associated edits from the five readers in the reader performance study

Figure 3 illustrates the model-generated impression that received the lowest average clinical accuracy along with the remainder of the report and edits from the panel of thoracic radiologist readers. We note the subjectivity in assigning a specific interstitial pneumonia pattern and the interplay between the stylistic preference of the attending radiologist including the addition and omission of certain findings.

**Fig. 4:**
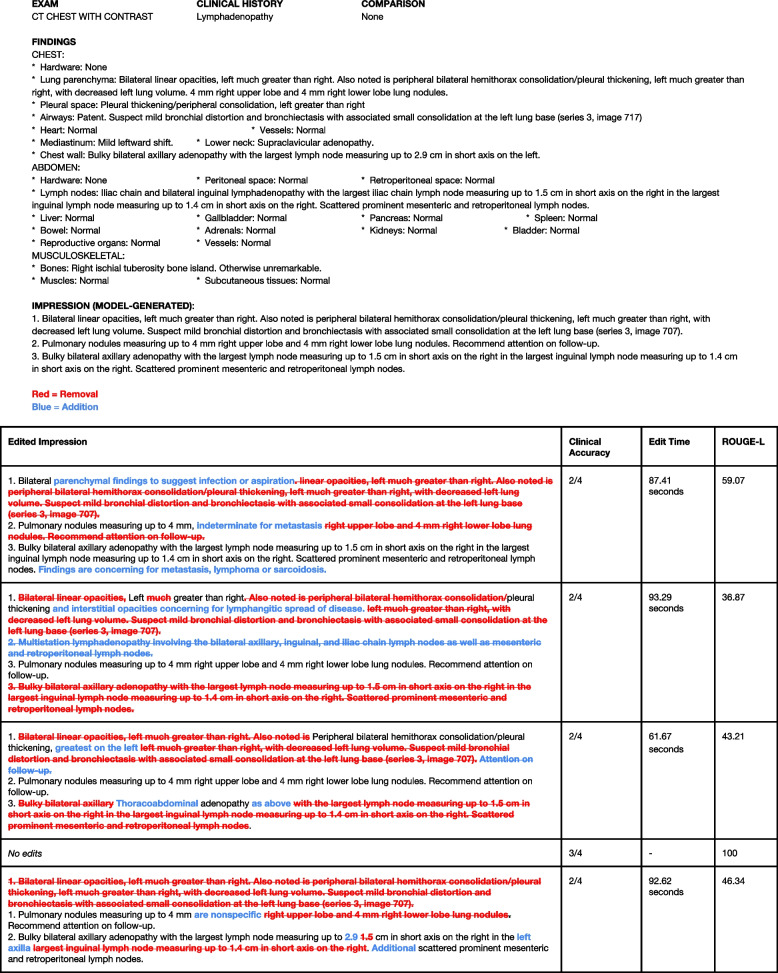
Lowest-scoring model-generated impression in terms of stylistic quality. Lowest-scoring model-generated impression in terms of stylistic quality and associated edits from the five readers in the reader performance study

Figure 4 illustrates the model-generated impression that received the lowest average stylistic quality. We note how the model tends to be verbose and include specific aspects of the findings section such as the size of the lymph node or note the particular series and slice that a finding is located, of which radiologists tend not to include the impression section. We also note the interplay between stylistic quality and clinical accuracy wherein the model failed to note if the findings are non-specific, or concerning for metastasis.

**Fig. 5:**
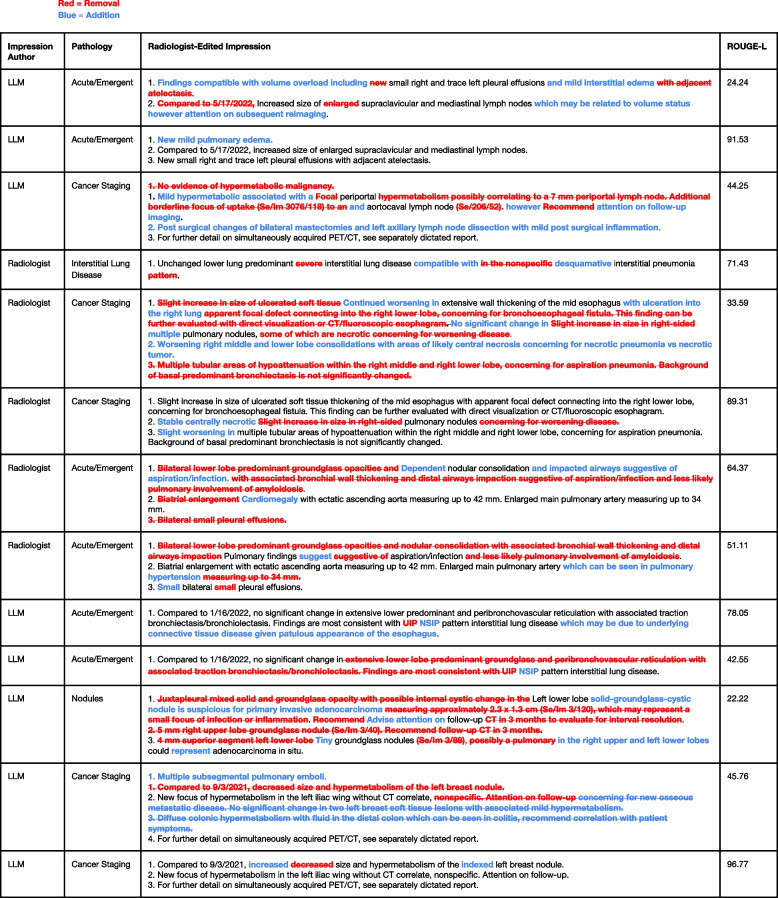
Radiologist edits for lowest clinical accuracy ratings in the reader performance study. Breakdown of edits for each impression, including both the model-generated and radiologist-written impressions, that received a rating of 1 out of 4 in terms of clinical accuracy. Reports shown multiple times reflect the edits of another reader

Figure 5 enumerates the modifications for every impression that received a rating of 1 out of 4 in terms of clinical accuracy from both model-generated impressions and radiologist-written impressions. This comprehensive breakdown illustrates a variety of clinical errors both from model-generated and radiologist-written impressions across different diagnosis categories.

**Fig. 6:**
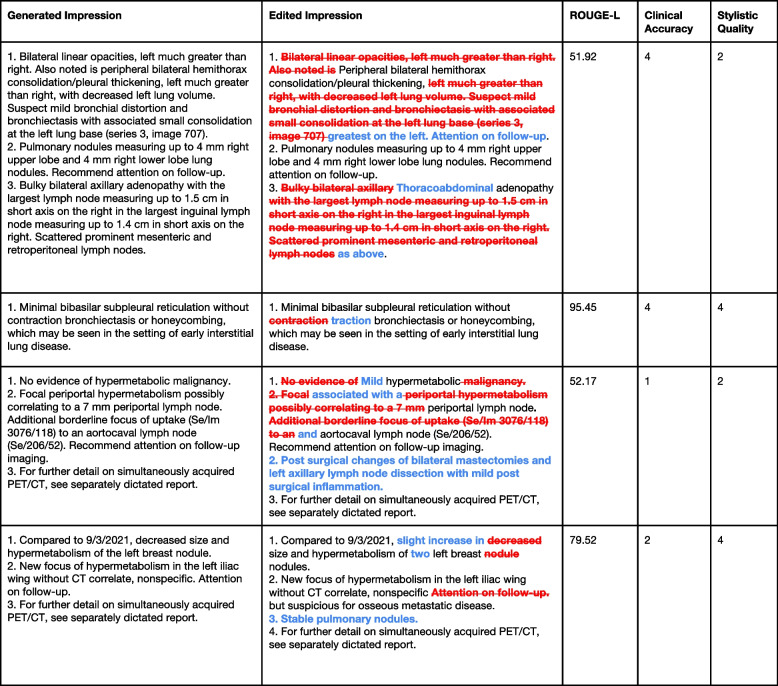
Sample cases from reader performance study with ROUGE scores. Sample cases that compare the ROUGE score across different pairs of generated impressions and their corresponding edits to better contextualize the ROUGE score in the clinical setting. A higher ROUGE score implies higher faithfulness to the reference impression

Figure 6 illustrates sample cases that compare the ROUGE score across different pairs of impressions. We note that ROUGE scores by definition measure adherence to the reference impression. We observe how ROUGE scores occasionally reflect stylistic quality better than clinical accuracy and note how it is integral to not rely on them and conduct reader performance studies to more reliably measure model performance.

## Discussion

We have evaluated a fine-tuned open-source large language model’s ability to generate impressions from the remainder of a radiology report over multiple imaging modalities and hospitals. On the UCSFMC test dataset, the LLM achieved ROUGE-1, ROUGE-2, and ROUGE-L scores of 53.22, 51.26, and 46.51 on CT reports, 51.26, 35.36, and 44.2 on MRI reports, and 56.41, 41.15, and 50.96, on US reports. We also tested the LLM’s performance on the ZSFG independent test set and it achieved scores of 46.57, 31.87, and 40.74 on CT reports, 45.04, 29.47, and 37.89 on MRI reports, and 32, 13.87, and 24.61, on US reports. For the reader performance study, the model-generated impressions achieved overall mean scores of 3.56/4, 3.92/4, and 3.37/4, 18.29 s, and 12.32 words for clinical accuracy, grammatical accuracy, stylistic quality, edit time, and edit distance respectively, while the original subspecialist radiologist impression baseline achieved overall mean scores of 3.75/4, 3.87/4, and 3.54/4, 12.2 s, 5.74 words respectively. Additionally, with respect to the readers’ edited impressions, the model-generated impressions achieved ROUGE-1, ROUGE-2, and ROUGE-L scores of 85 (82.89, 88.22), 81 (77.04, 84.41), and 84 (80.72, 87.13) respectively. On the other hand, the original impressions written by an attending radiologist achieved mean scores of 89 (85.96, 92.69), 85 (76.90, 89.30), and 89 (84.76, 92.31) respectively. The LLM achieved the highest clinical accuracy ratings for acute/emergent findings and on shorter impressions.

The ROUGE score results on the two hospital test datasets demonstrate a substantial overlap between the model-generated impressions and the original impression written by an attending radiologist. These scores may be impacted by the variability in writing impressions between radiologists, but act as a general gauge to assess potential model degradation in external validation. We sought to address this limitation in interpreting the ROUGE score by additionally conducting a reader performance study to more clinically assess if the model-written impression, though potentially different from the original radiologist’s impression, is of satisfactory quality. With respect to model edits in the reader study, the model had a substantially higher set of ROUGE scores, also evidenced by a relatively low edit distance to the revised indication written by the readers. This set of ROUGE scores demonstrates the potential to have LLMs preliminarily draft impressions that can be subsequently revised and finalized by radiologists. Overall, we note that the ROUGE scores can only be interpreted in relative terms, as the ROUGE scores for the automated lexical metrics measure the overlap of independently written impressions, while the reader study ROUGE scores are focused on the deviation from radiologists’ revisions on an already-written impression.

Our findings demonstrate the need to develop evaluation frameworks where automated lexical metrics are complemented by a reader performance study for a more comprehensive analysis of the generated impressions. Our reader performance study leads to a more granular and comprehensive analysis of the strengths and flaws of the large language model in generating impressions with a thoracic radiologist baseline. Aside from quantitative metrics such as clinical accuracy, grammatical accuracy, and stylistic quality, the reader study also examines impression quality with the radiologist’s word-for-word edits and edit time to simulate a workflow integrating large language models in radiology reporting. For instance, our stratified analysis by diagnosis reveals that the LLM performs best in terms of cancer staging and acute/emergent diagnosis categories, but slightly underperforms in terms of the Other category, including cases that included interstitial lung disease diagnosis categories. Particularly, for the impression that received the lowest average rating in terms of clinical accuracy, the radiologist readers noted how an impression generated by the model that mentions a UIP pattern instead of an NSIP pattern may adversely affect clinical care [23]. This finding on the clinical risks of LLMs has also been explored in other investigations that examined the use of LLMs for biomedical applications [24–26]. These error cases, despite few, demonstrate the necessity of radiologist supervision at this stage if it were to be integrated for clinical use.

Several studies have previously sought to automatically generate impressions using large language models. For instance, Sun et. al and Ma et. al have examined how to adapt GPT-4 to generate impressions for radiology reports [9, 22]. We build upon this body of work on automatic impression generation for radiology report summarization and focus on evaluating fine-tuned open-source large language models which would greatly enhance study replicability as opposed to closed-source models such as ChatGPT and GPT-4. Furthermore, the open-source nature of our study and full release of the associated code allows for further development in this area in contrast with the closed-source algorithms currently available in industry.

Our results present a framework for fine-tuning and evaluating an open-source large language model for automatic impression generation. Subsequent work in this area can focus on a prospective clinical validation of LLMs in enhancing the clarity and consistency of radiologist-written impressions, significantly improving the communication between physicians and radiologists. One such implementation could involve a hybrid approach of leveraging LLMs to draft radiology report impressions with subsequent revisions from radiologists with the resulting time-savings and reduction of costs from the streamlined workflow can be measured and evaluated.

Our study had several limitations. First of all, our automated lexical methodology of calculating the adherence of large language model output using the ROUGE score is not directly interpretable and can only be used in relative terms to gauge model performance (e.g. relative to other imaging modalities or hospital dataset). Second, our reader performance study only included sixty cases, due to the prohibitive cost and intractability of a large-scale reader study involving the manual editing and evaluation by subspecialist cardiothoracic radiologists. Our reader study was primarily intended to identify key areas where large language models can provide value in terms of generating impressions, but a more comprehensive analysis with a larger sample size and disease category stratification is deferred to future work. Third, only two hospitals that use the English language were included in the study which would imply that additional evaluation must be needed to establish the utility of the model to a broader clinical audience. Fourth, another methodical limitation is that given the scope of the study, we were unable to measure time savings in terms of absolute gain. To measure an unbiased estimate of the time taken for an attending radiologist to write an impression with and without this model, the large language model needs to be directly integrated into the clinical workflow via the dictation software requiring additional regulatory approval which we delegate to future work.

## Conclusions

In conclusion, we have evaluated a fine-tuned open-source large language model’s capacity to generate impressions for radiology reports across multiple imaging modalities and hospitals. Our reader performance study demonstrates that LLMs have the potential to greatly improve the workflow efficiency of radiologists by drafting preliminary versions of impressions and contribute to the quality of radiology reports.

## Data Availability

The data for this project came from UCSF radiology reports. The data can be shared with researchers using data use agreement and approval of the data committee at UCSF.

## Abbreviations

LLM: Large Language Model
ROUGE: Recall-Oriented Understudy for Gisting Evaluation
T5: Text-to-Text Transformer
